# Gambling refusal self-efficacy and protective behavioral strategies mediate and moderate the relationships between impulsivity and gambling outcomes

**DOI:** 10.1038/s41598-026-46112-w

**Published:** 2026-04-03

**Authors:** Natasa Nagy, Andrea Czakó, Zsolt Demetrovics, Zsolt Horváth

**Affiliations:** 1 Doctoral School of Psychology, ELTE Eötvös Loránd University, Budapest, Hungary; 2 Institute of Psychology, ELTE Eötvös Loránd University, Budapest, Hungary; 3 Flinders University Institute for Mental Health and Wellbeing, College of Human Sciences and Culture, Flinders University, Bedford Park, SA Australia; 4 Centre of Excellence in Responsible Gaming, University of Gibraltar, Gibraltar, Gibraltar

**Keywords:** Addictive behavior, Behavioral addictions, Gambling disorder, Impulsive behavior, Protective behavioral strategies, Refusal self-efficacy, Diseases, Neuroscience, Psychology, Psychology, Risk factors

## Abstract

Studies suggest that refusal self-efficacy and protective behavioral strategies may influence how impulsivity relates to addictive behaviors. However, their mediating and moderating roles have not yet been examined in the context of gambling. This study investigated whether gambling refusal self-efficacy (GRSE) and gambling protective behavioral strategies (GPBS) mediate or moderate the associations between impulsivity and gambling outcomes, including problem gambling (PG) and gambling involvement. A cross-sectional online survey was conducted among 926 gamblers from the general population (females: 53.2%; age: *M* = 41.85, *SD* = 13.49). Standardized self-report measures were used, including the Problem Gambling Severity Index, the Consumption Screen for Problem Gambling, the Barratt Impulsiveness Scale, the Gambling Self-Efficacy Questionnaire, and the Gambling Protective Behavior Scale. Linear regression-based mediation and moderation models were tested. Significant indirect associations between impulsivity and both PG and gambling involvement were observed via GRSE and GPBS. In addition, a significant interaction between impulsivity and GPBS indicated that associations between impulsivity and gambling outcomes were evident primarily at lower GPBS levels, whereas they were non-significant at higher levels. These findings extend prior evidence from other addictive behaviors to gambling, highlighting potential similarities across addictive processes. Longitudinal studies using representative or clinical samples are warranted.

## Introduction

Gambling disorder (GD) is a distinct addictive disorder, according to the fifth edition of the Diagnostic and Statistical Manual of Mental Disorders (DSM-5), characterized by diagnostic criteria such as the development of tolerance (e.g., increasingly larger bets are needed to achieve a state of excitement), restlessness and irritability when reducing/stopping gambling (withdrawal symptoms), multiple unsuccessful attempts to stop (loss of control), or impairment in several important areas of the gambler’s life (e.g., work, family)^1^. The symptoms and harms arising as a result of gambling can also be conceptualized as a spectrum of severity, referred to as problem gambling (PG). At the lower end of the spectrum are those who experience some symptoms without significant negative consequences and/or do not meet the diagnostic criteria for GD, while at the higher end are those who meet the above-mentioned diagnostic criteria and live with GD^2^. Although higher levels of gambling involvement (in terms of e.g., gambling frequency, time spent gambling, or financial expenditure) alone do not necessarily imply GD or harm, they are often linked to a greater risk of GD or PG^3^. According to a recent meta-analysis, 8.7% of the adult population worldwide were identified as experiencing any levels of problems (i.e., at-risk gamblers with at least one negative consequence), while 1.41% were described as people, whose gambling causes multiple different problems^4^. To have a better understanding of gambling-related problems and risks, it is important to examine the cognitive, behavioral, and personality factors that may be linked to the severity of gambling involvement and PG among individuals who do not necessarily meet criteria for GD and may show lower severity levels within the PG spectrum.

Impulsivity, as a personality trait, is a frequently documented risk factor in relation to various addictive behaviors, and has been examined within several theoretical frameworks^5–7^. Generally, impulsivity can be characterized as a tendency to act immediately without considering the long-term and potential negative consequences^8^. Regarding PG, meta-analyses showed that impulsivity is associated with PG and GD, which means that individuals with higher impulsivity are at increased risk of PG or GD^9–11^. In addition, some studies have reported positive associations between impulsivity and various indicators of gambling involvement^12,13^. These positive associations with PG, GD, and gambling involvement have been observed across various facets of impulsivity, including impairments in decision-making or attentional control, as well as a tendency toward impulsive behavior during negative emotional states^11,12^. People with higher impulsivity may be less able to control gambling when exposed to cues, and may have a preference for immediate rewards despite the possible long-term negative consequences^14^. Furthermore, the role of impulsivity was also highlighted in GD typologies according to the pathways model, where the antisocial-impulsivist subtype is characterized by increased antisocial, sensation-seeking traits and gambling behavior is driven by the motivation to increase positive emotions, or reduce boredom^15–17^.

Although impulsivity itself can be a risk factor for GD and PG^18^, it is possible that there are other cognitive-behavioral constructs that may influence the relationship. According to Bandura’s social cognitive theory, refusal self-efficacy (RSE) can be one determining factor behind addictive behaviors, and it may also be an important construct in understanding the relationship between addictive behaviors and impulsivity^19^. RSE is defined as one’s belief about their ability to resist engaging in certain behaviors, and several studies have shown its relationship with a range of addictive behaviors, including problematic alcohol and cannabis use^20–22^. Accordingly, gambling refusal self-efficacy (GRSE) refers to one’s confidence in refusing gambling in high-risk situations (e.g., being under the influence of other substances, experiencing negative emotions, etc.)^23^. Previous research showed that individuals characterized by lower GRSE are more likely to exhibit more severe levels of GD and PG, as well as greater gambling involvement (e.g., higher gambling frequency)^23–25^.

Another important factor can be one’s use of protective behavioral strategies (PBS). PBS are a series of conscious actions used to reduce harm related to an addictive behavior or to minimize/completely avoid negative consequences^26^. Regarding gambling, these strategies may be separated into two main factors: (1) harm reduction strategies (e.g., limiting the amount of time or money) and (2) avoidance strategies (e.g., minimizing engagement in gambling)^27,28^. Previous studies have indicated negative relationships between gambling-related protective behavioral strategies (GPBS) and both PG and gambling involvement (e.g., frequency, financial expenditure), suggesting that greater use of GPBS is linked to lower levels of gambling-related problems and involvement^28^.

As shown above, GRSE and GPBS can act as protective factors for PG, as they both can strengthen the control over gambling behavior (e.g., by avoiding gambling or by reducing the intensity and frequency of gambling). Thus, higher GRSE and greater use of GPBS may represent correlates of lower PG severity and gambling involvement. Although only a few studies have examined the relationship between impulsivity, GRSE, and GPBS, findings suggest negative associations, indicating that those with higher impulsivity tend to report lower GRSE and GPBS levels^29,30^. Studies in other addictive behaviors show that RSE and PBS can influence the associations between impulsivity and problematic alcohol^31^ and cannabis use^32^. On that note, it is possible that GRSE and GPBS may mediate and moderate the association between impulsivity and both PG and gambling involvement. Specifically, higher levels of impulsivity may be linked to lower GRSE and less frequent GPBS use, which in turn may be associated with higher levels of PG and gambling involvement^31–33^. Alternatively, GRSE and GPBS may moderate the association between impulsivity and both PG and gambling involvement, such that these associations may be stronger at lower levels of GRSE and GPBS and weaker at higher levels^34^. However, to the best of our knowledge, the complex relationships among these constructs, as well as the potential mediating and moderating roles of GRSE and GPBS in the associations between impulsivity, PG and gambling involvement, have not been examined. Investigating these associations may contribute to a better understanding of the processes linking impulsivity to gambling outcomes and may inform future longitudinal research.

### Study aims

The aim of the present study was to examine the potential mediating and moderating roles of GRSE and GPBS on the relationship between impulsivity and both PG and gambling involvement. Based on previous research on other addictive behaviors^31–33^, we hypothesized that GRSE and GPBS mediate the associations between impulsivity and both PG and gambling involvement. In other words, higher levels of impulsivity may be linked to lower GRSE and less frequent GPBS, which in turn may be linked to higher levels of PG and gambling involvement. In addition, we hypothesized that GPBS and GRSE moderate the associations between impulsivity and both PG and gambling involvement. Specifically, these associations may be stronger at lower levels of GPBS and GRSE and weaker (or potentially non-significant) at higher levels.

## Methods

### Procedures

An online, cross-sectional survey was conducted to investigate motivational, cognitive, and emotional factors, as well as personality traits, associated with PG. Participants were recruited through convenience sampling between April and May 2024 via the social media platform Facebook, using paid advertisements that appeared in the newsfeeds of potential participants to facilitate broad access to the target population of Hungarian adult past-year gamblers. To encourage participation, respondents were offered the opportunity to enter a prize draw for shopping vouchers. Participation in the draw was optional and not required for taking part in the study. Participation in the study itself was voluntary, with the option to withdraw at any time. Survey responses were collected without personally identifiable information. Those participants who wished to enter the prize draw were asked to provide an email address; these contact details were handled confidentially, stored separately from the survey data, and could not be linked to individual responses. Prior to beginning the questionnaire, all participants provided informed consent. The study protocol was reviewed and approved by the Research Ethics Committee of the Faculty of Education and Psychology, Eötvös Loránd University (no. 2023/422-2). The study was conducted in accordance with the principles outlined in the Declaration of Helsinki and relevant local regulations.

### Participants

A total of 1920 participants provided consent and began completing the questionnaire. However, participants were excluded from the final sample if they were younger than 18 years, had not gambled in the past 12 months, exited the survey before completion (i.e., did not reach the closing page of the questionnaire package), completed the survey in an unrealistically short time (i.e., the mean response time per item was below a threshold of two seconds), and provided responses with a high proportion of missing data (i.e., ≥ 75%). Based on these criteria, the final sample consisted of 926 participants. The demographic and gambling-related characteristics of the sample are presented in Table 1. A slightly higher proportion of women was represented in the sample compared to men, and the mean age of the sample was 41.85 years (*SD* = 13.49). Approximately two-thirds of participants were employed full-time. More than half of the sample reported mainly online gambling, while nearly one-third primarily engaged in land-based gambling. Gambling frequency showed a distribution in which roughly one-third reported gambling once a month or less frequently, another one-third reported gambling two to four times per month, and approximately one-third reported gambling multiple times per week. According to the Problem Gambling Severity Index (PGSI), nearly half of the sample fell into the non-problem gambling category, more than one-quarter were classified as low-risk problem gamblers, and approximately one-quarter were classified as moderate- or high-risk gamblers.

**Table 1:** Demographic and gambling-related characteristics of the sample.

**Demographic characteristics**	
Female sex	*N* = 493 (53.2%)
Mean age (*SD*)	*M* = 41.85 (13.49)
**Employment**	
Works full-time	*N* = 626 (67.6%)
Works part-time	*N* = 40 (4.3%)
Works occasionally	*N* = 69 (7.5%)
Unemployed	*N* = 191 (20.6%)
**Preferred modality of gambling**	
Mainly online (e.g., online betting or casino websites accessed via mobile phone or computer)	*N* = 503 (54.3%)
A mix of online and land-based	*N* = 130 (14.0%)
Mainly land-based (e.g., lottery shops, casino)	*N* = 293 (31.6%)
**Frequency of gambling in the past 12 months**	
Once a month or less frequently	*N* = 307 (33.2%)
Two to four times per month	*N* = 338 (36.5%)
Two to three times per week	*N* = 151 (16.3%)
Four or more times per week	*N* = 130 (14.0%)
**Risk of problem gambling based on the PGSI**	
Non-problem gambler	*N* = 421 (45.5%)
Low-risk problem gambler	*N* = 268 (28.9%)
Moderate-risk problem gambler	*N* = 159 (17.2%)
High-risk problem gambler	*N* = 77 (8.3%)

PGSI: Problem Gambling Severity Index.

### Measures

#### Problem Gambling Severity Index (PGSI)

PG was measured by the Problem Gambling Severity Index (PGSI)^35,36^. The PGSI is a nine-item questionnaire assessing gambling-related behavior and problems during the past 12 months with responses provided on a four-point scale (0 = Never, 1 = Sometimes, 2 = Often, 3 = Almost always). The total score ranges between 0 and 27 points, with higher scores reflecting greater PG severity. Based on the total score, participants can be categorized into subgroups: (1) non-problem gamblers (0 point); (2) low-risk problem gamblers (1–2 points); (3) moderate-risk problem gamblers (3–7 points); (4) high-risk problem gamblers (8 points or above). Excellent level of internal consistency was shown in the present study (*α* = 0.93).

#### Consumption Screen for Problem Gambling (CSPG)

Gambling involvement was measured using the brief, three-item Consumption Screen for Problem Gambling (CSPG)^37^. This brief screening instrument assesses key aspects of gambling involvement over the past 12 months, including gambling frequency (0 = No gambling in the past 12 months, 5 = Six or more times a week), typical time spent gambling (0 = Less than 30 min, 4 = More than three hours), and the frequency of extended gambling sessions exceeding two hours (0 = Never, 4 = Daily or almost daily). Responses to the first item are typically provided on a six-point scale. However, because participants reporting no gambling activity in the past 12 months were excluded, only five response categories were effectively used (i.e., item scores 1–5). The remaining two items use five-point response scales. The total CSPG score was used in the analyses. In the present sample, scores ranged from 1 to 13, with higher scores indicating greater gambling involvement. High internal consistency was observed in the present study (*α* = 0.81).

#### Barratt Impulsiveness Scale Revised (BIS-R-21-SF)

Impulsivity was measured by the short version of the Barratt Impulsiveness Scale – Revised^38–40^. The BIS-R-21-SF consists of 10 items that were derived from the original 21-item questionnaire (BIS-R-21). It includes three subscales: cognitive (4 items) and behavioral impulsivity (3 items), and impatience/restlessness (3 items). In addition, a total score can be calculated on the BIS-R-21-SF to assess overall impulsivity. In line with prior studies using the BIS-R-21-SF, the total score was used in the analyses in the present study^38,41^. Participants provided answers on a four-point scale about how often the given statements apply to them (1 = Never, 2 = Sometimes, 3 = Often, 4 = Almost always). The total score of the BIS-R-21-SF ranges between 10 and 40 points, with higher scores indicating higher impulsivity. Internal consistency was adequate in the current study (*α* = 0.78).

#### Gambling Self-Efficacy Questionnaire (GSEQ)

GRSE was measured using the Gambling Self-Efficacy Questionnaire (GSEQ)^42^. As part of the current study, the questionnaire was translated into Hungarian, back-translated into English, and subsequently reviewed by comparing the original and back-translated versions to finalize the Hungarian translation. The GSEQ is a 16-item scale assessing one’s belief to resist gambling in potentially high-risk situations (e.g., social pressure, emotional distress). Participants answered each item on a six-point scale (0 = Not at all confident [0%], 5 = Extremely confident [100%]). The total score of the GSEQ was calculated (ranging between 0 and 80 points), where higher scores indicate higher confidence in resisting gambling across high-risk situations (i.e., higher overall GRSE). Excellent level of internal consistency was shown in the present study (*α* = 0.95).

#### Gambling Protective Behavior Scale (GPB-S)

GPBS were measured by the Gambling Protective Behavior Scale (GPB-S)^28^. The Hungarian translation of the questionnaire was developed as part of the present study, following procedures similar to those applied for the GSEQ. The GPB-S is a 16-item questionnaire including two subscales: avoidance strategies (7 items) and harm reduction strategies (9 items). In addition, a total score can be computed to assess the overall level of GPBS. In the present study, this total score was used in the analyses, consistent with some of the previous studies applying this scale^30^. Participants rated the frequency of their use of GPBS in the last 12 months on a 5-point scale (0 = Never, 1 = Rarely, 2 = Sometimes, 3 = Most of the time, 4 = Always). Higher scores on the total scale (ranging between 0 and 64 points) indicate more frequent use of GPBS. The internal consistency was excellent in the present study (*α *= 0.88).

### Data analysis

For examining the bivariate relationships between these constructs, correlation analyses were conducted. As multiple variables (e.g., PGSI, GSEQ) did not show a normal distribution, Spearman’s rho correlations were calculated. Separate multiple linear regression-based mediation and moderation analyses were conducted to test the proposed mediation and moderation models. In these models, impulsivity served as the predictor variable, GRSE and GPBS were included as either mediators or moderators, and PG and gambling involvement were examined as outcome variables. The effects of sex (females vs. males), age, and preferred gambling modality (mainly online, mainly land-based, mixed) were also controlled as it was hypothesized that these variables can be associated with the level of PG^4,43,44^. For the mediation analysis, the robust maximum likelihood (MLR) estimation method was used because the assumption of normality was violated for multiple variables. Moderation analyses were conducted using ordinary least squares (OLS) multiple linear regression. Two separate models were estimated for PG and gambling involvement, with bootstrap resampling applied to obtain robust confidence intervals for the regression coefficients. Each model included four interaction terms (impulsivity × GRSE, impulsivity × GPBS, GRSE × GPBS, and impulsivity × GRSE × GPBS). The predictor and moderator variables were mean-centered prior to analysis. Correlation and mediation analyses were performed in JASP 0.95.2.0. The moderation analyses were conducted in IBM SPSS Statistics 25 using Andrew Hayes’ PROCESS macro (Model 3)^45^.

## Results

### Bivariate correlations

The results of the correlation analyses are presented in Table 2. Impulsivity was significantly and weakly to moderately correlated with lower levels of GPBS and GRSE, and higher levels of PG and gambling involvement. In addition, GPBS had weak but significant negative associations with PG and gambling involvement, and a weak but significant positive association with GRSE. A significant, moderate negative association was observed between GRSE and both PG and gambling involvement. In addition, a significant, strong positive correlation was observed between PG and gambling involvement.

**Table 2:** Bivariate correlations between the variables.

	1.	2.	3.	4.	5.
1. Impulsivity	-	-	-	-	-
2. Gambling protective behavioral strategies	-0.229***	-	-	-	-
3. Gambling refusal self-efficacy	-0.291***	0.263***	-	-	-
4. Problem gambling	0.305***	-0.234***	-0.456***	-	-
5. Gambling involvement	0.145***	-0.238***	-0.360***	0.521***	-
*M* (*SD*)	19.62 (4.72)	36.76 (14.00)	60.42 (19.68)	2.28 (4.14)	3.13 (2.73)
Min-Max	10–39	0–64	0–80	0–27	1–13

Level of significance: * *p* < 0.05, ** *p* < 0.01, *** *p* < 0.001. The correlation coefficients are Spearman’s rhos. *M* (*SD*): mean (standard deviation).

### Mediation analysis

**Fig. 1:**
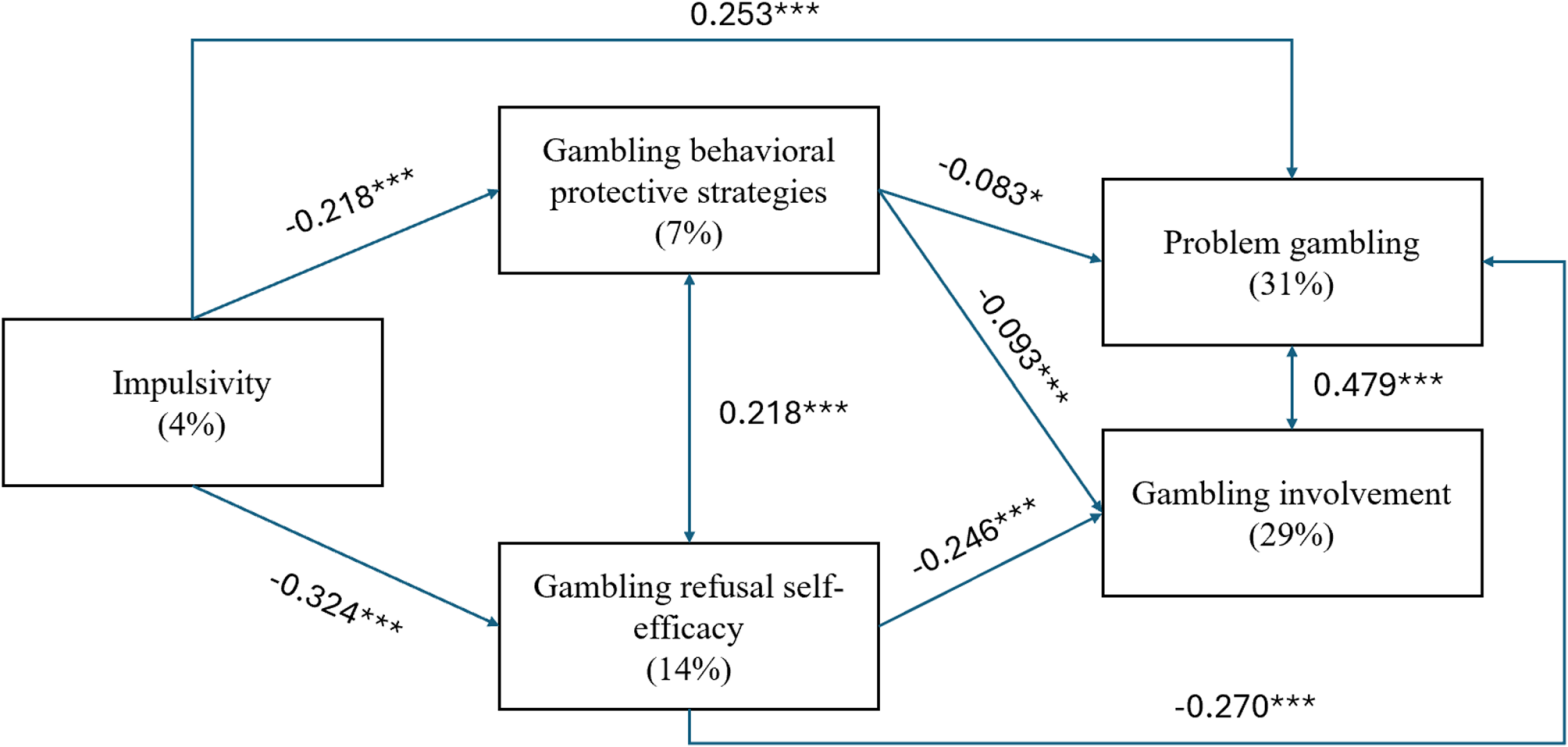
Significant pathways of the mediation model. Estimates in each pathway are standardized regression coefficients (*β*). The effects of age, sex and preferred gambling modality were also controlled in the model, but these are not shown to ease the interpretation of the findings (see: Table 3). Values in parentheses next to the name of each variable indicate the proportion of explained variance (*R*^*2*^). Level of significance: * *p* < .05, ** *p* < .01, *** *p* < .001.

Several significant associations were identified in the mediation model, which are summarized in Table 3; Fig. 1. To enhance the interpretability of the findings, only associations relevant to the study hypotheses are reported in the text; associations involving background covariates are not discussed here (see Table 3). Impulsivity was weakly negatively associated with GRSE and moderately negatively associated with GPBS, and was also weakly positively associated with PG; however, no significant association was observed with gambling involvement. Both GRSE and GPBS showed significant, weak negative associations with PG and gambling involvement. These significant relationships emerged after controlling for sex, age, and preferred gambling modality.

**Table 3:** Regression effects in the mediation model.

Predictor		Outcome	*β*	*SE*	*p*
Male sex (vs. female sex)	→	Impulsivity	0.039	0.035	0.259
Age	→	Impulsivity	-0.171	0.031	< 0.001
Preferred modality: Mixed (vs. Land-based)	→	Impulsivity	0.076	0.037	0.038
Preferred modality: Online (vs. Land-based)	→	Impulsivity	-0.029	0.035	0.401
Male sex (vs. female sex)	→	Gambling refusal self-efficacy	-0.160	0.032	< 0.001
Age	→	Gambling refusal self-efficacy	-0.140	0.036	< 0.001
Preferred modality: Mixed (vs. Land-based)	→	Gambling refusal self-efficacy	-0.071	0.031	0.025
Preferred modality: Online (vs. Land-based)	→	Gambling refusal self-efficacy	-0.041	0.032	0.211
Impulsivity	→	Gambling refusal self-efficacy	-0.324	0.031	< 0.001
Male sex (vs. female sex)	→	Gambling protective behavioral strategies	-0.095	0.033	0.005
Age	→	Gambling protective behavioral strategies	-0.021	0.036	0.555
Preferred modality: Mixed (vs. Land-based)	→	Gambling protective behavioral strategies	-0.049	0.033	0.137
Preferred modality: Online (vs. Land-based)	→	Gambling protective behavioral strategies	-0.084	0.035	0.016
Impulsivity	→	Gambling protective behavioral strategies	-0.218	0.035	< 0.001
Male sex (vs. female sex)	→	Problem gambling	0.119	0.028	< 0.001
Age	→	Problem gambling	-0.119	0.027	< 0.001
Preferred modality: Mixed (vs. Land-based)	→	Problem gambling	0.060	0.030	0.045
Preferred modality: Online (vs. Land-based)	→	Problem gambling	0.118	0.031	< 0.001
Impulsivity	→	Problem gambling	0.253	0.040	< 0.001
Gambling refusal self-efficacy	→	Problem gambling	-0.270	0.030	< 0.001
Gambling protective behavioral strategies	→	Problem gambling	-0.083	0.030	0.005
Male sex (vs. female sex)	→	Gambling involvement	0.229	0.027	< 0.001
Age	→	Gambling involvement	-0.023	0.027	0.385
Preferred modality: Mixed (vs. Land-based)	→	Gambling involvement	0.129	0.029	< 0.001
Preferred modality: Online (vs. Land-based)	→	Gambling involvement	0.268	0.029	< 0.001
Impulsivity	→	Gambling involvement	0.039	0.034	0.253
Gambling refusal self-efficacy	→	Gambling involvement	-0.246	0.031	< 0.001
Gambling protective behavioral strategies	→	Gambling involvement	-0.093	0.027	< 0.001

*β*: standardized regression coefficients. *SE*: standard error.

Estimates of the direct, indirect and total effects are summarized in Table 4. Two significant indirect associations were observed for PG. First, impulsivity was positively associated with PG indirectly through GRSE, accounting for 24% of the total effect. Second, a significant indirect association was also found through GPBS, accounting for 5% of the total effect. Similarly, two significant indirect associations were identified for gambling involvement, reflecting indirect links via GRSE and GPBS, accounting for 58% and 14% of the total effect, respectively.

**Table 4:** Direct, indirect and total effects of the mediation model.

	*β*	*SE*	*p*
**Direct effects**			
Impulsivity → Problem gambling	0.253	0.040	< 0.001
Impulsivity → Gambling involvement	0.039	0.034	0.253
**Indirect effects**			
Impulsivity → Gambling refusal self-efficacy → Problem gambling	0.087	0.012	< 0.001
Impulsivity → Gambling protective behavioral strategies → Problem gambling	0.018	0.007	0.009
Impulsivity → Gambling refusal self-efficacy → Gambling involvement	0.080	0.012	< 0.001
Impulsivity → Gambling protective behavioral strategies → Gambling involvement	0.020	0.007	0.004
**Total effects**			
Impulsivity → Problem gambling	0.358	0.041	<. 001
Impulsivity → Gambling involvement	0.139	0.035	< 0.001

*β*: standardized effect size. *SE*: standard error.

### Moderation analyses

Findings of the moderation analyses are shown in Tables 5 and 6. To enhance interpretability, associations involving background covariates are not discussed here (see Table 5). Impulsivity was not significantly associated with gambling involvement but showed a significant positive association with PG. GRSE was significantly and negatively associated with both PG and gambling involvement. GPBS showed a significant negative association with gambling involvement, whereas its association with PG was not significant. Among the four tested interaction terms, only the interaction between impulsivity and GPBS was significant, and this was observed for both PG and gambling involvement (Table 5). First, the association between impulsivity and gambling involvement was significant and positive at low levels of GPBS (with low and moderate levels of GRSE), whereas this association was non-significant at moderate and high levels of GPBS. Second, the association between impulsivity and PG was significant and positive at low and moderate levels of GPBS, irrespective of GRSE levels, but became non-significant at high levels of GPBS (Table 6).

**Table 5:** Regression effects in the moderation models.

	Outcome: Gambling involvement (*R*^2^ = 31%)		Outcome: Problem gambling (*R*^2^ = 38%)	
	*B*_Bootstrap_	95% *CI*_Bootrstap_	*B*_Bootstrap_	95% *CI*_Bootrstap_
Male sex (vs. female sex)	**1.2518**	**0.9376; 1.5627**	**1.0233**	**0.5251; 1.4829**
Age	-0.0043	-0.0156; 0.0067	**-0.0360**	**-0.0535; -0.0201**
Preferred modality: Mixed (vs. Land-based)	**0.9645**	**0.5134; 1.4241**	0.4071	-0.2368; 1.0799
Preferred modality: Online (vs. Land-based)	**1.5500**	**1.1681; 1.9381**	**0.9607**	**0.4176; 1.5148**
Impulsivity	0.0103	-0.0274; 0.0471	**0.1864**	**0.1231; 0.2532**
Gambling refusal self-efficacy	**-0.0276**	**-0.0368; -0.0189**	**-0.0415**	**-0.0545; -0.0289**
Gambling protective behavioral strategies	**-0.0172**	**-0.0300; -0.0050**	-0.0179	-0.0376; 0.0009
Impulsivity × Gambling refusal self-efficacy	0.0007	-0.0012; 0.0025	-0.0042	-0.0084; 0.0000
Impulsivity × Gambling protective behavioral strategies	**-0.0030**	**-0.0057; -0.0004**	**-0.0082**	**-0.0139; -0.0029**
Gambling refusal self-efficacy × Gambling protective behavioral strategies	0.0006	0.0000; 0.0013	0.0005	-0.0006; 0.0016
Impulsivity × Gambling refusal self-efficacy × Gambling protective behavioral strategies	0.0001	0.0000; 0.002	0.0001	-0.0001; 0.0004

*B*_Bootstrap_: Mean unstandardized regression coefficient estimated from 5000 bootstrap samples. 95% *CI*_Bootstrap_: 95% confidence intervals for the unstandardized regression coefficients based on bootstrap resampling. Regression coefficients were considered significant (*p* < 0.05) when the 95% bootstrap confidence interval did not include zero; significant coefficients are shown in bold. Regression coefficients were rounded to four decimal places to facilitate interpretation of whether confidence intervals included zero.

**Table 6:** Moderating effects of gambling refusal self-efficacy and gambling protective behavioral strategies on the association between impulsivity and gambling outcomes.

Gambling refusal self-efficacy level^1^	Gambling protective behavioral strategies level^2^	Outcome: Gambling involvement			Outcome: Problem gambling		
		*B*	*SE*	*p*	*B*	*SE*	*p*
Low	Low	0.060	0.025	0.019	0.418	0.037	< 0.001
Low	Moderate	-0.003	0.027	0.902	0.266	0.039	< 0.001
Low	High	-0.066	0.043	0.119	0.113	0.062	0.069
Moderate	Low	0.053	0.025	0.035	0.301	0.036	< 0.001
Moderate	Moderate	0.010	0.019	0.584	0.185	0.027	< 0.001
Moderate	High	-0.032	0.026	0.208	0.070	0.037	0.061
High	Low	0.046	0.035	0.196	0.183	0.051	< 0.001
High	Moderate	0.024	0.026	0.359	0.105	0.038	0.005
High	High	0.002	0.032	0.955	0.027	0.047	0.560

^1^The moderator variable was mean-centered; low, moderate, and high levels were defined as one standard deviation below the mean (-19.74), the mean (0.00), and one standard deviation above the mean (19.73), respectively. ^2^The moderator variable was mean-centered; low, moderate, and high levels were defined as one standard deviation below the mean (-13.97), the mean (0.00), and one standard deviation above the mean (13.97), respectively. *B*: unstandardized regression coefficient representing the association between impulsivity and the respective outcome variable. *SE*: standard error.

## Discussion

The aim of the present study was to examine the potential mediating and moderating roles of GRSE and GPBS on the relationship between impulsivity and both PG and gambling involvement. Based on previous research in the field of other addictive behaviors^31–33^, we hypothesized that GRSE and GPBS would mediate the associations between impulsivity and PG and gambling involvement, such that higher impulsivity would be associated with lower levels of GRSE and GPBS, which in turn would be associated with higher levels of PG and gambling involvement. In addition, we hypothesized that GPBS and GRSE would moderate the associations between impulsivity and both PG and gambling involvement. It was expected that the associations would be stronger at lower levels of GPBS and GRSE and weaker, or potentially non-significant, at higher levels.

The findings of the mediation analysis supported our hypotheses, both GRSE and GPBS mediated the associations between impulsivity and both PG and gambling involvement, while controlling for sex, age, and preferred gambling modality. However, due to the cross-sectional nature of this study, these indirect associations must be interpreted with caution, as the study does not provide evidence of causal relationships. Significant indirect associations via GRSE were observed for both PG and gambling involvement, indicating that higher impulsivity was associated with lower GRSE levels, which in turn were associated with higher levels of both outcomes. These associations are consistent with previous research showing that lower levels of GRSE are associated with impulsivity^29^, PG, and gambling involvement (i.e., frequency)^25^. In addition, previous studies on alcohol drinking^31^ and cannabis use^32^ have shown that substance-specific RSE mediates the relationship between different impulsivity dimensions (e.g., negative urgency) and problematic use of these substances. Thus, individuals with higher levels of impulsivity may show behavioral and attentional biases toward stimuli offering immediate gratification (e.g., the prospect of large gambling-related winnings and the excitement associated with it), which may be associated with reduced attention to potential negative consequences of gambling (e.g., debt)^14^. Consequently, they may feel less confident in resisting gambling in high-risk situations (e.g., social pressure, positive or negative emotions), which in turn may be linked to higher rates of gambling-related problems and negative consequences.

Significant indirect associations via GPBS were observed for both PG and gambling involvement, indicating that higher impulsivity was associated with lower GPBS use, which in turn was associated with higher levels of both outcomes. These results are in line with previous research showing a negative relationship between GPBS and impulsivity^30^, as well as between GPBS and both PG and gambling involvement^28^. The potential mediating role of PBS is well-documented in relation to impulsivity in other addictive behaviors, including problematic alcohol^33^ and cannabis use^32^. This pattern suggests that individuals with higher impulsivity traits may be less likely to apply either avoidance strategies (e.g., avoiding venues related to gambling) or harm reduction strategies (e.g., limiting time or money) which in turn may be associated with higher levels of gambling involvement and gambling-related problems and negative consequences.

Although significant indirect associations via GRSE and GPBS were identified, the direct association between impulsivity on PG remained significant, indicating partial mediation. In contrast, no significant direct association was observed between impulsivity and gambling involvement, with the indirect associations accounting for a relatively larger proportion of the total association compared with PG. The robust association between impulsivity and PG – persisting even after controlling for sex, age, and preferred gambling modality – is in line with prior research^31–33^. Furthermore, these findings are consistent with previous research emphasizing that impulsivity is more closely associated with PG than with gambling involvement^46^. Previous research also suggests distinct associations of impulsivity dimensions with these outcomes, with trait impulsivity (conceptually closer to the BIS-R-21-SF used in the present study) associated with PG, whereas sensation seeking (not assessed in the present study) has been associated with gambling involvement^47^.

The findings of the moderation analyses provided only partial support for the proposed hypotheses. A significant interaction between GPBS and impulsivity was observed for both PG and gambling involvement, whereas the other tested interaction terms were non-significant, including those involving GRSE. The associations between impulsivity and both PG and gambling involvement were significant and positive at low levels of GPBS, and for PG also at moderate levels, whereas these associations were non-significant at high levels of GPBS. In addition, the strength of the associations between impulsivity and both PG and gambling involvement decreased with increasing levels of GPBS. These results are aligned with previous research suggesting a moderating role of PBS in addictive behaviors (e.g., problem drinking), with the strength of associations between impulsivity and addictive outcomes varying across PBS dimensions and levels^34^. Overall, the present findings might suggest that less frequent use of GPBS may reflect conditions under which the positive associations between impulsivity and gambling outcomes may become more pronounced.

It is also important to consider that, in addition to GRSE and GPBS, other constructs may also mediate or moderate the associations between impulsivity and both PG and gambling involvement, such as gambling motivations^48^, anxious-depressive symptoms^49^, or emotion regulation strategies^32^. For example, a previous study examining the mediating roles of RSE and PBS in relation to anxious-depressive symptoms suggests that negative affective states may also be relevant to the associations between impulsivity, GRSE, GPBS, and gambling outcomes. Associations involving lower self-regulation capacities, both general and gambling-specific (i.e., lower GRSE and GPBS), may be more pronounced in the presence of negative affective states and less adaptive emotion regulation strategies in high-risk situations^32^. Alternatively, indirect associations between impulsivity and gambling outcomes may also involve enhancement motives (i.e., gambling to experience positive emotions)^48^. However, the potential roles of these additional moderator and mediator variables could not be examined within the scope of the present study.

Finally, although background confounding variables were not part of the main hypotheses, they warrant brief consideration given several significant associations with gambling-related outcomes. Male sex was significantly associated with higher levels of both gambling involvement and PG, and with lower levels of both GRSE and GPBS. Additionally, younger age was significantly associated with higher PG and lower GRSE, but not with gambling involvement or GPBS. These findings are consistent with prior research showing that male sex and younger age are positively associated with PG, and that male sex is also associated with lower GRSE and avoidance strategy-related GPBS^4,28,42–44,50^. In contrast, previous findings regarding sex differences in gambling involvement indicators (e.g., frequency, expenditure) have been more inconclusive^51^, and prior studies have also reported non-significant associations between age and GRSE^42,52^. Overall, the association of male sex with less favorable gambling-related outcomes, as well as the relationship between younger age and PG and GRSE, may reflect the joint contribution of sociocultural, psychological, and biological factors (e.g., masculinity norms, novelty seeking, and risk-taking)^43^.

Preference for mainly online gambling was associated with higher gambling involvement and PG, as well as lower GPBS, while a preference for mixed gambling modality was linked to lower GRSE. These findings may align with earlier research indicating that the presence of at least some form of online gambling (i.e., mixed or mainly online modality) is associated with more harmful and problematic gambling outcomes, which may reflect structural characteristics commonly attributed to online gambling environments, such as constant availability and easier, more continuous access^44,53,54^.

### Limitations

There were multiple limitations to the present study that might have biased the findings. First, a cross-sectional design was used; therefore, causal connections cannot be interpreted between the variables. It is possible that there are bidirectional relationships between the abovementioned variables, which could not be examined in the present study. For example, impulsivity might not only be an antecedent factor regarding PG, but the accumulation of gambling-related problems and negative consequences may lead to impulsive, rash behavior. Second, self-report questionnaires were applied, which may have biased the reliability of responses, for example, due to social desirability bias, memory bias, or lack of self-awareness. Additionally, although some questionnaires (i.e., GSEQ, GPB-S) were translated into Hungarian for this study, full psychometric validation of these versions was beyond the scope of the present research. Furthermore, impulsivity was assessed using a brief version of the BIS, primarily to reduce participant burden, as its short length (10 items) helped limit response fatigue during questionnaire completion. The BIS has also been widely used in research examining associations between impulsivity and gambling-related outcomes, supporting comparability with previous findings^9,38^. While the intention was to capture a global self-reported impulsivity construct, this choice limited the examination of specific impulsivity dimensions in relation to PG and gambling involvement, for which multidimensional measures (e.g., the short UPPS-P) may have been more suitable^55^. Third, a non-representative sample of gamblers was recruited, thus the present findings cannot be generalized to the Hungarian gambler population. It is possible that due to the applied social media-based recruitment, male gamblers, those with limited access to the internet, and land-based gamblers were less likely to be included in the sample. Finally, due to the nature of the sample (i.e., general population of gamblers), the proportion of high-risk gamblers was low, thus it is possible that different results would have been demonstrated on a clinical sample (participants with GD) or a sample consisting only of problem gamblers. However, it is also important to note that, compared with previous studies using the PGSI in representative adult samples, the proportion of non-problem gamblers (i.e., individuals reporting no gambling-related harms or problems in the past 12 months) was lower in the present sample, whereas higher proportions were observed for the low-, moderate-, and high-risk problem gambling categories^56–58^.

## Conclusions

The present study aimed to test the mediating and moderating roles of GRSE and GPBS in the associations between impulsivity and PG and gambling involvement. First, the results supported the hypotheses regarding the proposed mediation model, with significant indirect associations via GRSE and GPBS. Second, partial support was found for the moderation hypotheses, as a significant impulsivity × GPBS interaction indicated that associations between impulsivity and gambling outcomes were evident primarily at lower GPBS levels but not at higher levels. Overall, these patterns resemble those reported in other addictive behaviors, suggesting potential similarities across addictive processes^31,33,34^. In addition, these findings may have potential clinical relevance and implications. For example, the use of cognitive behavioral therapeutic techniques such as cognitive restructuring or behavior substitution aimed at increasing GRSE or GPBS may help one decrease the possibility of engaging in gambling in risky situations and may be useful for at-risk individuals^59,60^.

Several directions for future research can be proposed, which are also in alignment of more general focuses of gambling research priorities^61^. First, it would be informative to carry out additional cross-sectional studies using representative or clinical samples to gain better understanding of differences between subgroups of gamblers^62^. Second, due to the bias of self-report questionnaires, it would be beneficial to use other assessment methods, such as clinical interviews, registering daily diaries, and behavioral tests of impulsivity. Third, longitudinal studies are needed to investigate the causal pathways between impulsivity and PG. For example, applying an intensive longitudinal design (e.g., experience sampling method, ecological momentary assessment) would be beneficial to observe momentary changes between the variables^63^. Finally, it would be also useful to conduct research on the impact of clinical interventions which aim to increase GRSE and the use of GPBS^59,60^.

## Data Availability

Data supporting the findings of this study are available from the corresponding author upon reasonable request.
